# Embryo placement in IVF and reproductive outcomes: a cohort analysis and review

**DOI:** 10.5935/1518-0557.20190003

**Published:** 2019

**Authors:** Marcella Melo dos Santos, Adelino Amaral Silva, Antonio César Paes Barbosa, Guilherme Brum, Hitomi Miura Nakagawa, Iris Cabral, Jose Rubens Iglesias, Marina Wanderley Paes Barbosa

**Affiliations:** 1 GENESIS - Center for Assistance in Human Reproduction, Brasília, DF, Brazil

**Keywords:** embryo placement, reproductive outcome, IVF

## Abstract

**Objective:**

The objective of the present study was to determine the influence of the embryo placement depth on the endometrial cavity in relation to the reproductive outcomes, after frozen-thawed embryo transfer performed under transabdominal ultrasound guidance.

**Methods:**

Retrospective cohort study that evaluated the influence of the embryo placement depth in the endometrial cavity in relation to the reproductive outcomes of patients submitted to cryotransfer cycles at a private assisted reproduction clinic, from 2012 to 2017. The patients were classified according to three variables: <10mm, 10 to 15mm and >15mm. The primary outcome was clinical pregnancy, and the secondary outcomes were miscarriage, ongoing pregnancy and live birth. The data was summarized as relative risk, with a 95%CI.

**Results:**

Clinical and ongoing pregnancy rates were higher in the 10-15mm and >15mm Groups, when compared to the <10mm Group; there was no statistical difference between the groups in terms of miscarriage and live birth rates. We performed a subsequent analysis, using the same sample of patients, comparing only the <10mm and ≥10mm variables. The ≥10mm Group had better reproductive outcomes, with higher clinical and ongoing pregnancy rates.

**Conclusion:**

Pregnancy rates are influenced by embryo transfer site, and better results can be achieved when the tip of the catheter is placed in the central area of the endometrial cavity, especially when the distance from the endometrial fundus is >10mm.

## INTRODUCTION

A new age started after the birth of the first baby from In Vitro Fertilization (IVF) in 1978. Over the past three decades, assisted reproductive technologies (ART), including IVF and intracytoplasmic sperm injection (ICSI), have proven to be promising and useful for most infertile couples (Tiras *et al*., 2010). Female age, ovarian reserve, embryo quality, endometrial receptivity, and embryo transfer techniques can be considered determinants of IVF treatment success, thus predicting reproductive outcomes (Özcan et al., 2016).

Success rates with ART have improved significantly in the last decades. Despite major advances in ovarian stimulation protocols and in embryo culture media, few improvements have been reported in embryo transfer (ET) techniques. While the average birth rate associated with ART was 28% in 1996, it increased to 35.4% in 2006. Embryo transfer, the final stage of ART, has acquired special importance as a crucial step in ensuring a successful IVF (Friedman *et al*., 2011), with most recent studies focusing on providing a standardization and improvement in techniques (Özcan et al., 2016).

One of the main determinants of embryo transfer is operator’s ability to deposit the embryos where the chances of implantation are higher, without causing trauma to the endometrium (López *et al*., 2014). The ideal embryo transfer site has been debated by several researchers. The air bubbles loaded into the catheter with the embryo (s) could be considered a marker to determine the final position of the embryo (s) in the uterine cavity (Özcan et al., 2016). Studies evaluating the ideal depth of embryo transfer in the uterine cavity have reported that fundus transfers are associated with higher pregnancy rates (PRs), but there is still no consensus in these regards (Confino *et al*., 2007).

The objective of the present study was to determine the influence of the embryo placement depth on the endometrial cavity in relation to reproductive outcomes, after frozen-thawed embryo transfer performed under transabdominal ultrasound guidance (Tiras *et al*., 2010).

## MATERIALS AND METHODS

The present study was a retrospective cohort study that evaluated the influence of the placement depth of the embryos in the endometrial cavity in relation to reproductive outcomes of patients submitted to cryotransfer cycles at a private assisted reproduction clinic - Genesis, located in Brasília, Federal District, from 2012 to 2017. All patients underwent endometrial preparation with Estradiol Valerate (LV) at a fixed dose of 6 mg/day or increasing doses, with the addition of progesterone (natural micronized in the vaginal capsule formula 600 mg/day, vaginal gel 8%-90mg/day or dydrogesterone 30 mg/day) when the endometrium reached the trilaminar aspect and minimum thickness of 7.0mm.

All cryotransfers were performed by experienced operators using the Wallace catheter (Smiths Medical International Ltd.) and transabdominal ultrasonography as a guide for visualization. All procedures were performed without anesthesia or sedation. The women were placed in the lithotomy position, with a moderately full bladder. The cervix was exposed through a vaginal speculum, followed by vaginal cleansing with 0.9% saline and cervical mucus removal with sterile cotton swab. The embryos were loaded into the catheter and then transferred to the uterine cavity, with microscopic verification by the embryologist immediately afterwards, to assure that no embryo remained in the catheter. The distance from the air bubble to the uterine fundus was measured by ultrasonography immediately after ET. At this point, the patients in whom it was not possible to see the bubble, or it was not described, were excluded from the cohort.

We included embryo transfers in cleavage (D3) and blastocyst (D5/D6) stages. Those patients submitted to endometrial preparation with natural and modified natural cycles were excluded, as well as those who were using transdermal VE. The patients were randomly assigned according to the distance between the air bubble (reference site to be considered as embryo deposition site) and the uterine fundus at the time of transfer, and were classified according to three variables: ≤10mm, 10 to 15mm and >15mm.

Biochemical pregnancy was determined by the quantitative BhCG test performed 12 days after embryo transfer. Clinical pregnancy was defined as ultrasound evidence of embryo with a heartbeat, at a gestational age (GA) of 6 weeks; miscarriage was defined as fetal loss with GA <20 weeks and / or fetal weight <500g; ongoing pregnancy was defined as gestation with GA >20 weeks, but still in course; live birth was considered as the product of a birth in which there was evidence of life at birth. The primary endpoint was to assess clinical pregnancy. Secondary outcomes were ongoing pregnancy, live birth, and miscarriage rates.

This study was approved by the clinical board, with exemption from the informed consent term and submission to the Ethics Committee, since this was a retrospective study analyzing medical records.

The statistical analysis was carried out using the RevMan 5.3 software; the measures were analyzed as relative risk (RR), with a 95% confidence interval (CI). A value of *p*<0.05 was considered significant.

## RESULTS

A total of 264 patients were analyzed, of which 56 were excluded from the study for incomplete or absent information, and 208 were included in the sample. The mean age of the patients was 35.55 years with a standard deviation of 5.93 years. Reproductive outcomes are summarized in Table 1.

**Table 1:** Reproductive outcomes in the three groups studied

	DISTANCE		
	<10mm (n=61)	10-15mm (n=123)	>15mm (n=19)
Clinical pregnancy	21% (13/61)^a,b^	43% (53/123)^a^	58% (11/19)^b^
Miscarriage	31% (4/13)	30% (16/53)	36% (4/11)
Ongoing pregnancy	15% (9/61)^c,d^	30% (37/123)^c^	37% (7/19)^d^
Live Birth	10% (6/61)	15% (19/123)	26% (5/19)

*p*-values: a = 0.008; b = 0.001; c = 0.03; d = 0.03

Clinical pregnancy rates were higher in the 10-15mm and >15mm Groups, when compared to the <10mm Group RR 2.02 [CI 95% 1.20, 3.41] and RR 2.72 [CI 95% 1.47, 5.03], respectively); there was no difference between the groups 10-15mm and >15mm Groups (RR 0.74 [CI 95% 0.48, 1.15]).

Similarly, the ongoing pregnancy rate was higher in the 10-15mm and >15mm Groups in comparison with the <10mm Group (RR 2.04 [CI 95% 1.05, 3.95] and RR 2.50 [CI 95% 1.07, 5.80], respectively); there was no difference between the 10-15mm and >15mm Groups (RR 0.82 [CI 95% 0.43, 1.56]).

There was no statistical difference between the groups in terms of miscarriage and live birth rates.

Considering these results and trying to establish a safe distance in which the embryo placement generates a greater success rate, we performed a subsequent analysis, using the same sample, comparing only the <10mm and ≥10mm variables, as shown in Table 2. The ≥10mm Group displayed better reproductive outcomes, with higher clinical pregnancy rates (RR 2.11 [CI 95% 1.26, 3.54]) and ongoing pregnancy rates (RR 2.10 [CI 95%1.10, 4.03]). Miscarriage and live birth rates were similar in both groups.

**Table 2:** Reproductive outcomes in the two groups studied

	DISTANCE	
	<10mm (n=61)	≥10mm (n=142)
Clinical pregnancy	21% (13/61)^a^	45% (64/142)^a^
Miscarriage	31% (4/13)	31% (20/64)
Ongoing pregnancy	15% (9/61)^b^	31% (44/142)^b^
Live Birth	10% (6/61)	17% (24/142)

*p*-values: a = 0.004; b = 0.03

## DISCUSSION

Embryo transfer is the final step in ART, so it should be performed with caution, since it may represent a variable that significantly affects pregnancy rates. Embryo transfer can be performed in three ways: (1) blindly (clinical touch); (2) based on information on uterine length, obtained by ultrasonographic measurement or simulated transfer; (3) guided by abdominal ultrasonography. It is advantageous to place the embryos in the uterus as atraumatically as possible. Uterine contractions, presence of blood or mucus in the catheter, bacterial contamination of the catheter, difficulty in transfer, embryo exposure to environmental conditions, and catheter type, can all influence the success rate of an IVF/ICSI treatment. A high success rate has been reported with the abdominal ultrasound guidance technique in the presence of a full bladder, ensuring that the embryos are being placed in the proper site and avoiding direct contact with the uterine fundus, which causes contractions. Another factor that dramatically affects the rates of implantation is the experience of the doctor who performs it. There is some controversy whether the site of embryo placement can be an important variable in the embryo transfer technique (Pacchiarotti *et al*., 2007).

It was traditionally accepted that embryos should be placed at approximately 10 mm below the surface of the endometrial fundus (Pacchiarotti *et al*., 2007). According to Cenksoy *et al*. (2014), the ideal position of the embryos would be at a distance of <10mm from the endometrial fundus. Lambers *et al*. (2007) believed that the position of air bubbles after ET is related to PR, and the highest rates were found when the air bubbles occupied the region closer to the uterine fundus. A retrospective cohort study designed by Friedman *et al*. (2011), including 315 blastocyst transfers suggested that placement closer to the endometrial fundus surface (<10mm) is associated with a higher PR.

On the other hand, Pacchiarotti *et al*. (2007) showed a significant increase in pregnancy and implantation rates in the group in which the embryo was placed at a distance of 10 to 15mm between the tip of the catheter and the uterine fundus. Similarly, Tiras *et al*. (2010), in a retrospective analysis with 5,055 embryo transfers, deduced that the distance > 10mm and <20mm may be the best site for embryo placement, reaching higher PRs and that, in addition to 1 cm from the bottom, the placement of embryos anywhere within the cavity has no negative effect on PRs. This study further mentions that the common final conclusion of some studies is that embryo transfer at a distance less than or equal to 10mm from the endometrial fundus was associated with a lower pregnancy rate compared to transfers at more than 10mm of the final surface of the endometrium. According to a guideline (Practice Committee of the American Society for Reproductive Medicine, 2017), based on the common ASRM practice, there is evidence based on seven studies (three RCTs and four cohort studies) that placement of the catheter tip in the upper or middle (central) area of the uterine cavity, at more than 1cm from the uterine fundus optimizes pregnancy rates (Grade B evidence).

However, some researchers have suggested that placement of embryos lower into the endometrial cavity and further away from the background may improve pregnancy rates in IVF/ICSI cycles (Cavagna *et al*., 2006). Coroleu *et al*. (2002) concluded that a fixed distance of 15 to 20 mm from the endometrial fundus surface can optimize embryo transfer performance compared to a 10 mm insert. In addition, pregnancy rates are significantly higher when the site selected is approximately 2cm from the uterine fundus. Frankfurter *et al*. (2003) demonstrated better pregnancy rates when the embryos were placed further from the uterine fundus. Frankfurter *et al*. (2004) concluded that higher rates of implantation and pregnancy are obtained when embryo transfers are performed in the mid or lower uterus segments compared to the upper segment. Pope *et al*. (2004) performed a multivariate logistic regression analysis on 699 embryo transfers, which showed that for every additional millimeter of embryo placement farther from the endometrial fundus, the odds of clinical pregnancy increase by 11%.

Nikas *et al*. (1995), studying endometrial biopsies, emphasized the presence of pinopods as markers of the “fertile window” located at 2cm from the uterine fundus. However, IVF cycles involve situations in which the endometrium undergoes stimuli that do not occur in the natural process. These lead to a localized or generalized premature decomposition in animal models, which may lead to the closure of the fertile window in vivo (Frankfurter *et al*., 2003). On the other hand, some studies, like the one from Levi Setti *et al*. (2003), showed that embryos must be placed in the middle of the cavity, away from the bottom. In a randomized study, Franco *et al*. (2004), placed the embryos in the lower or upper half of the endometrial cavity, and they reported no difference in pregnancy or implantation rates. Oliveira *et al*. (2004) obtained better results when the tip of the catheter was positioned near the central area of the endometrial cavity. They concluded that the relative site of embryo placement is more important than the actual distance from the uterine fundus. An RCT (Kwon *et al*., 2015) showed no difference in implantation or pregnancy rates when the tip of the catheter was placed 2 cm from the uterine fundus or in the middle third of the cavity, further supporting embryo placement in the upper or middle portion of the uterine cavity.

After transfer, the embryo is thought to be situated between the area where the catheter tip is located and the area where air bubbles spread immediately after transfer (Liedholm *et al*., 1980; Krampl *et al*., 1995; Baba *et al*., 2000; Cavagna *et al*., 2006). This is likely to be the region where the embryo will be implanted (Lambers *et al.*, 2007). Cavagna *et al*. (2006) concluded that when embryos are transferred to the center of the endometrial cavity, there is an increase in the rate of implantation in the central region compared to naturally conceived pregnancies. However, a successfully implanted embryo can be found in a different area than expected for different reasons. According to Tiras & Cenksoy (2014), there is a movement of the air bubbles soon after the placement of the embryos in the uterine cavity that can influence the rates of implantation and pregnancy. The movement of the embryos towards the cervical canal may be associated with lower clinical pregnancy rates, whereas movement towards the uterine fundus may be associated with higher pregnancy rates. In addition, there are those who did not find any association. The study by Kovacs *et al*. (2012) found no impact of the transfer site on the implantation rate or ongoing pregnancy rates.

Our study found a trend towards an unfavorable treatment outcome when the distance of embryo placement is less than 10mm from the uterine fundus. It is also evident that the highest rates of clinical pregnancy occur at a distance between 10 and 15mm, similar to some studies, without statistical relevance at a distance greater than 15mm from the endometrial fundus. In order to establish a safe distance for embryo deposition, we chose to reduce the variables (greater or less than 10mm), and the analysis showed a significant association between clinical pregnancy, ongoing pregnancy and live birth rates and the distance of embryo deposition. The occurrence of a favorable outcome is associated when this distance is greater than 10mm from the uterine fundus, which is also supported by some well-designed studies. One of the limitations of our study was the fact that the cryotransfers were performed by several professionals, who, although skilled, can influence the final results of implantation. The size of the sample with only 208 analyzed transfers is another factor that limits our findings.

Finally, in spite of the different conclusions on the subject, the position of the air bubbles in embryo transfer was considered relevantly associated with pregnancy rates, but, at present, it is not possible to predict or control this position with accuracy. After placing the transfer catheter, the final position of the air bubbles will depend on the syringe, the plunger resistance, the pressure used on the plunger, factors related to the patient, such as a possible intrauterine resistance, uterine contractions during ET, as well as a transfer considered easy and atraumatic. Therefore, we feel that there is a need for a standard method of embryo transfer that enables the evaluation of the exact embryo position (Lambers *et al.*, 2007).

Results of current studies demonstrate, in agreement with our study, that pregnancy rates are influenced by the embryo transfer site, with better results when the tip of the catheter is placed in the central area of the endometrial cavity, especially when the distance from the endometrial fundus is >10mm.

## References

[r1] Baba K, Ishihara O, Hayashi N, Saitoh M, Taya J, Kinoshita K (2000). Where does the embryo implant after embryo transfer in
humans?. Fertil Steril.

[r2] Cavagna M, Contart P, Petersen CG, Mauri AL, Martins AM, Baruffi RL, Oliveira JB, Franco Jr JG (2006). Implantation sites after embryo transfer into the central area of
the uterine cavity. Reprod Biomed Online.

[r3] Cenksoy PO, Ficicioglu C, Yesiladali M, Akcin OA, Kaspar C (2014). The importance of the length of uterine cavity, the position of
the tip of the inner catheter and the distance between the fundal
endometrial surface and the air bubbles as determinants of the pregnancy
rate in IVF cycles. Eur J Obstet Gynecol Reprod Biol.

[r4] Confino E, Zhang J, Risquez F (2007). Air bubble migration is a random event post embryo
transfer. J Assist Reprod Genet.

[r5] Coroleu B, Barri PN, Carreras O, Martínez F, Parriego M, Hereter L, Parera N, Veiga A, Balasch J (2002). The influence of the depth of embryo replacement into the uterine
cavity on implantation rates after IVF: a controlled, ultrasound-guided
study. Hum Reprod.

[r6] Franco Jr JG, Martins AM, Baruffi RL, Mauri AL, Petersen CG, Felipe V, Contart P, Pontes A, Oliveira JB (2004). Best site for embryo transfer: the upper or lower half of the
endometrial cavity. Hum Reprod.

[r7] Frankfurter D, Silva CP, Mota F, Trimarchi JB, Keefe DL (2003). The transfer point is a novel measure of embryo
placement. Fertil Steril.

[r8] Frankfurter D, Trimarchi JB, Silva CP, Keefe DL (2004). Middle to lower uterine segment embryo transfer improves
implantation and pregnancy rates compared with fundal embryo
transfer. Fertil Steril.

[r9] Friedman BE, Lathi RB, Henne MB, Fisher SL, Milki AA (2011). The effect of air bubble position after blastocyst transfer on
pregnancy rates in IVF cycles. Fertil Steril.

[r10] Kovacs P, Sajgo A, Rarosi F, Kaali SG (2012). Does it really matter how far from the fundus embryos are
transferred?. Eur J Obstet Gynecol Reprod Biol.

[r11] Krampl E, Zegermacher G, Eichler C, Obruca A, Strohmer H, Feichtinger W (1995). Air in the uterine cavity after embryo transfer. Fertil Steril.

[r12] Kwon H, Choi DH, Kim EK (2015). Absolute position versus relative position in embryo transfer: a
randomized controlled trial. Reprod Biol Endocrinol.

[r13] Lambers MJ, Dogan E, Lens JW, Schats R, Hompes PG (2007). The position of transferred air bubbles after embryo transfer is
related to pregnancy rate. Fertil Steril.

[r14] Levi Setti PE, Albani E, Cavagna M, Bulletti C, Colombo GV, Negri L (2003). The impact of embryo transfer on implantation-a
review. Placenta.

[r15] Liedholm P, Sundstrom P, Wramsby H (1980). A model for experimental studies on human egg
transfer. Arch Androl.

[r16] López MJ, García D, Rodríguez A, Colodrón M, Vassena R, Vernaeve V (2014). Individualized embryo transfer training: timing and
performance. Hum Reprod.

[r17] Nikas G, Drakakis P, Loutradis D, Mara-Skoufari C, Koumantakis E, Michalas S, Psychoyos A (1995). Uterine pinopodes as markers of the ‘nidation window’ in cycling
women receiving exogenous oestradiol and progesterone. Hum Reprod.

[r18] Oliveira JB, Martins AM, Baruffi RL, Mauri AL, Petersen CG, Felipe V, Contart P, Pontes A, Franco Júnior JG (2004). Increased implantation and pregnancy rates obtained by placing
the tip of the transfer catheter in the central area of the endometrial
cavity. Reprod Biomed Online.

[r19] Özcan P, Fıçıcıoğlu C, BalcıKokulu M, Alagöz O, Yesiladalı M (2016). Can the Movement of the Air Bubbles After Embryo Transfer Predict
the Success of IVF Treatment?. J Clin Anal Med.

[r20] Pacchiarotti A, Mohamed MA, Micara G, Tranquilli D, Linari A, Espinola SM, Aragona C (2007). The impact of the depth of embryo replacement on IVF
outcome. J Assist Reprod Genet.

[r21] Pope CS, Cook EK, Arny M, Novak A, Grow DR (2004). Influence of embryo transfer depth on in vitro fertilization and
embryo transfer outcomes. Fertil Steril.

[r22] Penzias A, Bendikson K, Butts S, Coutifaris C, Falcone T, Fossum G, Gitlin S, Gracia C, Hansen K, Mersereau J, Odem R, Rebar R, Reindollar R, Rosen M, Sandlow J, Vernon M, Practice Committee of the American Society for Reproductive
Medicine (2017). The ASRM Standard Embryo Transfer Protocol Template: A Committee
Opinion. Fertil Steril.

[r23] Tiras B, Polat M, Korucuoglu U, Zeyneloglu HB, Yarali H (2010). Impact of embryo replacement depth on in vitro fertilization and
embryo transfer outcomes. Fertil Steril.

[r24] Tiras B, Cenksoy PO (2014). Practice of embryo transfer: Recommendations during and
after. Semin Reprod Med.

